# *Pinus Roxburghii* essential oil anticancer activity and chemical composition evaluation

**DOI:** 10.17179/excli2016-670

**Published:** 2018-03-12

**Authors:** Arfaa Sajid, Qaisar Manzoor, Munawar Iqbal, Amit Kumar Tyagi, Raja Adil Sarfraz, Anam Sajid

**Affiliations:** 1 Government College Women University Faisalabad, Faisalabad, Pakistan; 2 Department of Chemistry, The University of Lahore, Lahore, Pakistan; 3 Cytokine Research Laboratory, Department of Experimental Therapeutics, The University of Texas M.D. Anderson Cancer Center, Houston, Texas, USA; 4 Department of Chemistry, University of Agriculture, Faisalabad, Pakistan; 5 Department of Chemistry, University of Punjab, Lahore, Pakistan

**Keywords:** Pinus roxburghii, anticancer agent, essential oil, NF-kappaB, MTT assay

## Abstract

The present study was conducted to appraise the anticancer activity of *Pinus roxburghii* essential oil along with chemical composition evaluation. MTT assay revealed cytotoxicity induction in colon, leukemia, multiple myeloma, pancreatic, head and neck and lung cancer cells exposed to essential oil. Cancer cell death was also observed through live/dead cell viability assay and FACS analysis. Apoptosis induced by essential oil was confirmed by cleavage of PARP and caspase-3 that suppressed the colony-forming ability of tumor cells and 50 % inhibition occurred at a dose of 25 μg/mL. Moreover, essential oil inhibited the activation of inflammatory transcription factor NF-κB and inhibited expression of NF-κB regulated gene products linked to cell survival (survivin, c-FLIP, Bcl-2, Bcl-xL, c-Myc, c-IAP2), proliferation (Cyclin D1) and metastasis (MMP-9). *P. roxburghii* essential oil has considerable anticancer activity and could be used as anticancer agent, which needs further investigation to identify and purify the bioactive compounds followed by *in vivo* studies.

## Introduction

The secondary metabolites of medicinal plant in crude extracts are well known for their bioactivity such as chemo preventive agents against cancer. In Asian countries historical background of medication shows that medicinal plants (products) have been utilized in cancer treatment (Abate et al., 2017[1]; Abebe et al., 2017[2]; Asif, 2015[5][7]; Cahyana et al., 2017[12]; Hamid et al., 2016[23]; Prasanna et al., 2011[41]; Yasir et al., 2016[53][54]). *Pinus roxburghii, *also known as chir pine, is native to Himalayas and distributed over a range of Himalaya range of Pakistan, Bhutan, Afghanistan, China, Nepal and southern India (Shuaib et al., 2014[47]). Five species of *Pinaceae* including *P. roxburghii *are found in Pakistan covering the total area of 1928 thousand hectares and mostly located in the rangelands of North West Frontier, Baluchistan and Punjab provinces (Hassan and Amjid, 2009[25]). In Asian sub-continent, *P. roxburghii* is traditionally used as a medicinal plant for the treatment of dermatological and topical diseases, gastrointestinal disorders, liver, spleen, ear, throat and skin, bronchitis, diaphoresis, giddiness, ulcer, inflammation, itching ailments and to cure snake bite (Sinha et al., 2013[48]). Various authors reported the anti-cancer activities of medicinal plants (Chouaïb et al., 2016[15]; Devi et al., 2015[16]; Heo et al., 2014[26]; Jose et al., 2016[30]; Khlifi et al., 2013[35]; Vuong et al., 2015[50]; Yessoufou et al., 2015[55]). Apart from traditional usage of *P. roxburghii*, the antidyslipidemic (Puri et al., 2011[42]), antioxidant (Qadir and Shah, 2014[43]; Salem et al., 2014[44]), anti-inflammatory, anticonvulsant, analgesic (Kaushik et al., 2012[32]), antiparasitic (Farooq et al., 2008[17]), antidiabetic (Kaushik et al., 2014[34]) and antimicrobial (Iqbal et al., 2011[28]) activities have also been reported. Recent studies also revealed the anticancer activities of some species of *Pinacea *family (Cho et al., 2014[14]; Jo et al., 2012[29]; Kaushik et al., 2015[33]; Yang et al., 2010[52]).

Chemical composition of *P. roxburghii *(needles, stem, bark and essential oil) have been studied well (Iqbal et al., 2011[28]; Qadir and Shah, 2014[43]; Satyal et al., 2013[46]) and caryophyllene, thunbergol, 3-carene, cembrene and α-pinene were the major constituents in *P. roxburghii *extracts (Hassan and Amjid, 2009[25]; Salem et al., 2014[44]). In view of considerable anticancer activities of *Pinacea *family plants, it will be of worth to study the anticancer activity of *P. roxburghii *plant. Therefore, the present investigation was conducted to appraise the anticancer potential of *P. roxburghii* leaves essential oil using different human cancer cell lines. Moreover, chemical composition of *P. roxburghii* essential oil (PREO) was also evaluated using advanced techniques. For anticancer activity evaluation of PREO, initially, different cell lines were used. After initial trails, two types of cells i.e., adherent (A549, HCT-116) and suspension (U-266, KBM-5) were selected. For colony forming assay adherent cancer cell lines line HCT 116 are required, whereas KBM-5 cell line was used to study apoptosis effect of PREO.

## Material and Methods

### Materials

Antibodies against cyclinD1, matrixmellatoproteinase-9 (MMP-9), PARP, cellular inhibitor of apoptosis 2 (c-IAP2), Bcl-2, Bcl-xl, c-Myc, β-actin and caspase-3 were obtained from Santa Cruz Biotechnology (USA). Survivin antibody and antibody against cellular FLICE-inhibitory protein (c-FLIP) were purchased from R & D Systems and Imgenex (San Diego, CA, USA), respectively. Secondary antibodies i.e., goat anti-rabbit and goat anti-mouse horseradish peroxidase conjugates were purchased from Bio-Rad (Hercules, CA, USA). Penicillin, streptomycin, DMEM, RPMI-1640, Iscove's DMEM and fetal bovine serum were obtained from Invitrogen (USA). 

### Cell lines

Human cancer cell lines i.e., HCT-116 (human colon cancer), KBM-5 (human myelogenous leukemia), U-266 (human multiple myeloma cells), MiaPaCa-2 (human pancreatic cancer cells), A-549 (human lung carcinoma cells), SCC-4 (squamous cell carcinoma) were obtained from the American Type Culture Collection. The HCT-116, MiaPaCa-2 and A-549 cells were cultured in DMEM, the U-266 cells were cultured in RPMI-1640 medium, whereas KBM-5 cells were cultured in Iscove's DMEM. DMEM and RPMI media were supplemented with 10 % fetal bovine serum (FBS), whereas Iscove's DMEM supplemented with 15 % FBS with 1 % antibiotics (100 U penicillin/mL and 100 mg streptomycin/mL) (Gibco, USA) was used. 

### 3-(4,5-Dimethylthiazol-2-yl)-2,5-diphenyltetrazolium bromide (MTT) cell proliferation assay

PREO effect on the proliferation of cancer cell was determined by measuring the mitochondrial dehydrogenase activity using MTT assay. This assay relies on the mitochondrial dehydrogenases of viable cells which cleave the tetrazolium ring of MTT and yields formazan form, which is monitored at 570 nm (ELISA micro plate reader, Bio-Tek, USA). Cells were seeded at a concentration of 5.0×10^3^ cells/0.1 mL in a 96-well plate for 12 h at 37 °C in a humidified incubator in the presence of 5 % CO_2_ (CO_2_ Incubator, Forma Scientific, USA). PREO was dissolved in DMSO and cells were treated in triplicate with 10, 25, 50 and 100 µg/mL oil concentrations (with this solution being added to the appropriate growth media to yield a final DMSO concentration of 0.1 % and final mixture was incubated for 72 h at 37 °C). Then, 20 µL MTT (5 mg/mL) solutions were added and again contents were incubated for 2 h at 37 °C. Finally, 100 µL buffers (cell lysis) was added and incubated overnight at 37 ^o^C. The optical density of the suspension was measured at 570 nm using MRX Revelation 96-well multiscanner (Dynex Technologies, Chantilly, VA) (Sankara et al., 2013[45]). The percentage residual cell viability was determined using relation shown in Eq. 1. Where, OD_s_ and OD_c_ are the optical densities of sample and control, respectively.


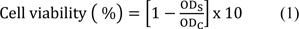


### Trypan blue exclusion assay

Cells were seeded in 96 well plates and treated with 25, 50 and 100 µg/mL PREO concentrations for 24 h at 37 °C and mixed with equal volume of isotonic trypan blue (0.4 %). Total cell number and fraction of nonviable, dye accumulating cells were counted after 2 min in Fuchs-Rosenthal hemocytometer under light microscope (Sung et al., 2013[49]).

### Clonogenic assay

Cells were grown and colonized by clonogenic assay. HCT-116 cells were seeded in 6-well plates 1.0×10^3 ^cells/well. After 24 h, cells were treated with essential oil 25, 50 and 100 µg/mL concentrations, incubated at 37 °C for 48 h and subjected to clonogenic assay as reported elsewhere (Gupta et al., 2013[19]). 

### Apoptosis assay

For determination of apoptotic effects of PREO on colon cancer cells, Live/dead assay kit (Molecular Probes, Eugene, OR) method was used, which determines the intracellular esterase activity and plasma membrane integrity. Intracellular esterases from live cells convert nonfluorescent, cell-permeable calceinacetoxy methyl ester to the intensely green fluorescent calcein, a polyanionic dye which was retained within cells. This assay was also used to examine the damaged membrane of cells (the ethidium bromide homodimer-1 enters damaged cells when bound to nucleic acids, it produces a bright red fluorescence). HCT-116 cells were seeded in 96-well plates 2.0×10^4^/well. After 12 h, cells were treated with 25, 50 and 100 µg/mL PREO concentrations for 24 h and then, washed with PBS (Gupta et al., 2011[20]). 

### Propidium Iodide (PI) staining for apoptotic cells

KBM-5 cells were seeded at 2×10^6 ^cells/1 mL in 6 well plates and incubated for 2 h at 37 °C, then treated with 10, 25 and 50 µg/ mL PREO for 24 h at 37 °C. Cells were harvested by centrifugation (2000 rpm, 1 min at 4 °C) and washed with PBS and re-suspended in 0.3 mL of PBS followed by 700 µL ethanol with constant slow stirring. After complete mixing, the content kept at -20 °C for 30 min, centrifuged and supernatant was decanted and cells were washed with PBS. The pellets obtained were re-suspended in 0.5 mL Propidium Iodide (PI) (1 mg/mL in PBS) and 5 µL of RNAse (Li et al., 2004[37]). 

### Western blot analysis

The PREO effect on protein expression was studied using KBM-5 cells following method reported elsewhere (Buhrmann et al., 2013[11]). In colony forming assay adherent cancer cell lines are required and HCT-116 is a type of adherent cell line, so far, it was for colony and apoptosis assay instead of KBM-5. Briefly, KBM-5 (2.0×10^6^) cells were seeded in 12-well plates for 2 h at 37 °C and treated with 10, 25, 50 and 100 µg/mL PREO concentrations for 24 h at 37 °C. Cells were harvested by centrifugation (14000 rpm for 30 sec at 4 °C), washed twice with cold PBS. Prepared cells mixed with cell lysis buffer (20 mM Tris, pH 7.4, 250 mM NaCl, 2 mM EDTA, pH 8, 0.1 % Triton X-100, 0.01 mg/ml aprotinin, 0.005 mg/ml leupeptin, 0.4 mM phenyl methyl sulfonyl fluoride, and 4 mM NaVO_4_) for 20 min. The contents were centrifuged for 10 min at 14000 rpm and supernatant was collected for further analysis. Protein contents were measured by Bradford assay. Proteins were separated on 10 % SDS-polyacrylamide gel electrophoresis (PAGE) and proteins were electro-transferred to nitrocellulose membranes using Trans-Blot apparatus (Bio-Rad Laboratories, Hercules, CA, USA) and blocked for 1 h in 5 % (w/v) skimmed milk powder in PBS/0.1 % Tween 20. Nitrocellulose membranes were incubated overnight (4 °C) with the primary antibody directed against MMP-9, cyclinD1, PARP, c-IAP2, Bcl-2, Bcl-xl, c-Myc, β-actin, c-FLIP, survivin and caspase-3 at a 1:3,000 dilution in blocking buffer at 4 °C on a shaker, washed thrice with washing buffer and then, incubated with secondary antibody conjugated with alkaline phosphatase for 90 min at ambient temperature. Membranes were washed five times. Specific antigen-antibody complexes were detected by enhanced chemiluminescence (Amersham Pharmacia Biotech, Piscataway, NJ) using ECL reagent (GE Healthcare).

### Electrophoretic Mobility Shift Assay (EMSA) for Nuclear Factor (NF)-κB

KBM-5 cells were seeded in 12-well plates (2.0×10^6 ^cells/1 mL), incubated for 2 h at 37 °C and treated with 10, 25 and 50 µg/mL PREO concentrations. After 8 h of incubation, cells were harvested and washed twice with cold PBS. Swelling induction in cell was induced by adding cold cytoplasmic extraction buffer and content was kept in ice for 15 min. The cytoplasmic cell fraction was lysed by adding 3.125 µL, 10 % Igepal (Sigma-Aldrich, St. Louis, MO)/100 µL CEB and mixed on vortex for 20 sec. Then, suspension was centrifuged for 5 min and cytosolic supernatant was discarded and cold nuclear extraction buffer was added to the pellet. Nuclear suspension was incubated for 30 min on ice (mixed the contents after every 5 min on vertex) and then, centrifuged at 10,000 rpm for 10 min. Nuclear protein concentration was determined by Bradford assay. The binding reaction was initiated by adding 15 µg nuclear extract to binding buffer (100 µM HEPES, pH7.9; 50 µMEDTA; 100 µMDTT; and 10 % glycerol), 2 µg poly (dI:dC) (Amersham Biosciences, Piscataway, NJ), 3.0 ×10^5^ counts per minute 32P-labeled 45-mer double-stranded NF-κB oligo nucleotide (15 μg of protein with 16fmol DNA) from the human immunodeficiency virus long terminal repeat (5′-TTGTTACAAGGGACTTTCCGCTGGGG ACTTTC CAGGGAGGCGTGG-3′) and 1 % Igepal (total volume, 20 µL) and incubated the mixture for 30 min at 37 °C. The reaction was terminated by adding 4 µL 6X DNA loading dye and sample was placed on ice and then, loaded on a pre-run 6.6 % polyacrylamide gel electrophoresis. A double-stranded mutated oligonucleotide (5′-TTGTTACAACTCACTTTCCGCTGCTCA CTTTCCAGGGAGGCGTGG-3′) was used to examine the specificity of binding of NF-κB to the DNA. The gel was dried and placed on film for autoradiography (Han et al., 2014[24]).

### Gas chromatography/mass spectrometry (GC-MS) analysis 

The PREO analysis was performed using GC-MS analysis (Agilent-Technologies, Little Falls, CA, USA) 6890N Network gas chromatographic (GC) system, equipped with an Agilent-Technologies 5975 inert XL Mass selective detector and Agilent-Technologies 7683B series auto injector used for oil analysis and GC-MS analysis performed as precisely reported (Hossain et al., 2012[27]). 

## Results and Discussion

**Figure 1:**
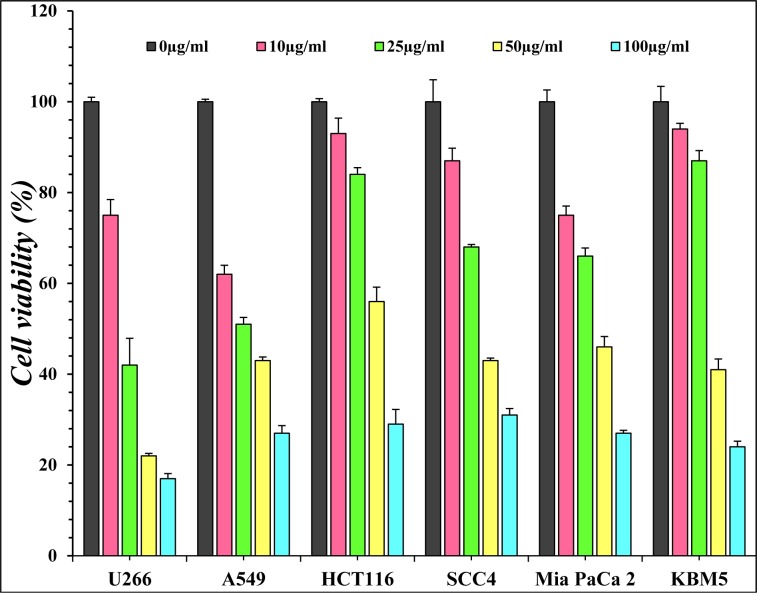
Effect of *Pinus roxburghii *on tumor cell proliferation. U266, A549, HCT16, SCC4, MiaPaCa2 and KBM-5 (5×10^3^ cells/well) were seeded in triplicate in 96-well plates; cells were treated in triplicate with (0, 10, 25, 50 and 100 μg/mL) concentrations of *Pinus roxburghii *essential oil (PREO) for 72 h, and then assayed for cell viability by the MTT method.

**Figure 2:**
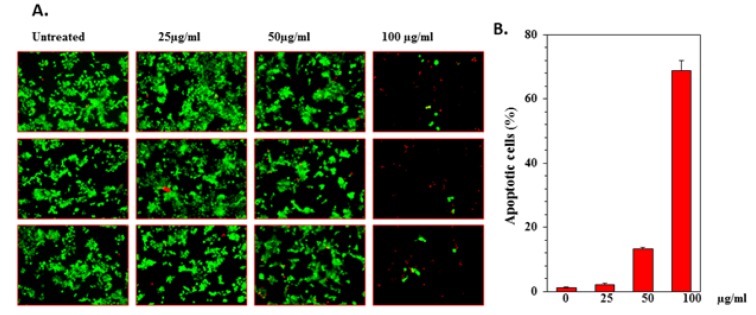
HCT-116 cells were seeded in 96-well plates 2.0×10^4^. After 12 h, cells were treated with (25, 50 and 100 μg/mL) concentrations of PREO for 24 h, removed the media and washed with PBS. Cells were then stained with assay reagents for 30 min at ambient temperature. Cell cytotoxicity was determined by live/dead assay. Data represent the mean±SD of three field measurements.

**Figure 3:**
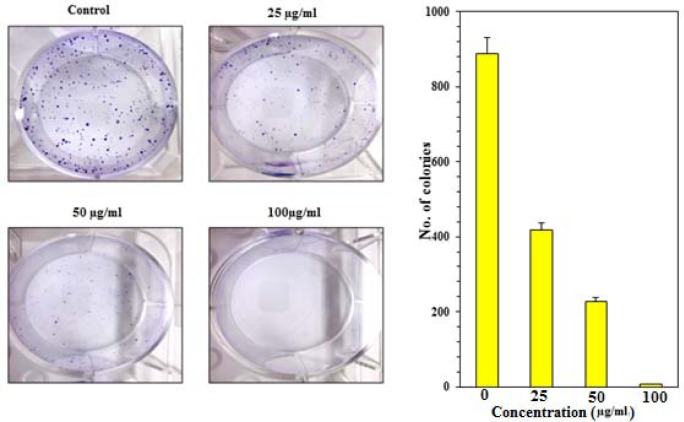
HCT-116 cells were seeded in 6-well plates 1.0 ×10^3^. After 24 h, cells were treated with (25, 50 and 100 μg/mL) concentrations of PREO. After 48 hours, the medium containing essential oil was washed off; the cells were allowed to form colonies for 12 days. Stained with 0.5 % crystal violet solution, washed once with PBS, air-dried, and counted.

PREO was studied against human cancer cell lines i.e., cultured HCT-116 (colon cancer), KBM-5 (myelogenous leukemia), U-266 (multiple myeloma cells), MiaPaCa-2 (pancreatic cancer cells), A-549 (lung carcinoma cells) SCC-4 (squamous cell carcinoma) cell lines by MTT assay. The percentage inhibition activity of PREO was found to be concentration-dependent (Figure 1(Fig. 1)). U-266 exhibited maximum inhibition of 83 %, while HCT-116, SCC4, MiaPaCa-2, A-549 and KBM-5 showed 71, 69, 73, 73 and 76 (%) inhibitions, respectively. These finding are in line with previous studies i.e., effect of *P. roxburghii* oil against A-549 (lung), C6 (glioma), T47D (breast), MCF-7 (breast) and TH-1(colon) cell lines by MTT assay that oil exhibits cytotoxicity against all tested cancer cells (Qadir and Shah, 2014[43]). In another study, needle and bark oil of *P. roxburghii* showed cytotoxicity against MCF-7 cell at 100 µg/mL (Satyal et al., 2013[46]). Similarly, petroleum, ether and chloroform extract of *P. roxburghii* also showed cytotoxicity against IMR-32 human neuroblastoma cell lines (Kaushik et al., 2012[32]). The cytotoxicity of PREO was correlated with high concentrations of terpinen-4-ol, (E)-caryophyllene, and α-humulene, which have been shown to be cytotoxic to MCF-7 cells (Wright et al., 2007[51]). Initially, different cell lines were used and the result showed that oil is effective against all cell lines. Then, the PREO oil activity against two types of cancer cell lines a) adherent (A549, HCT-116) and b) suspension (U-266, KBM-5) was tested (depending on the cell condition) because some assays perform good with adherent cell lines and some performed better with suspension cell lines (since assays are specific due to their adherent and suspension nature) (Gupta et al., 2016[21]; Kang et al., 2011[31]). So far HCT-116 cells were treated with different concentrations of PREO for 24 h and subjected to live/dead assay. The apoptotic cells percentages were 2.07, 13.22 and 68.87 (%) at 25, 50 and 100 µg/mL (Figure 2A, B(Fig. 2)), respectively. It was observed that PREO had less effect on colony formation of HCT-116 cells at 25 μg/mL, but it completely suppressed the colony-forming ability of tumor cells at 100 μg/mL concentration. The colonies were reduced from 417 to 6 as the concentration of PREO increased (Figure 3(Fig. 3)). Most of the studies were performed using the human myeloid cell line KBM-5 because these cells express tumor necrosis factor (TNF) receptors and the inflammatory pathway in these cells is well understood (Han et al., 2014[24]).

**Figure 4:**
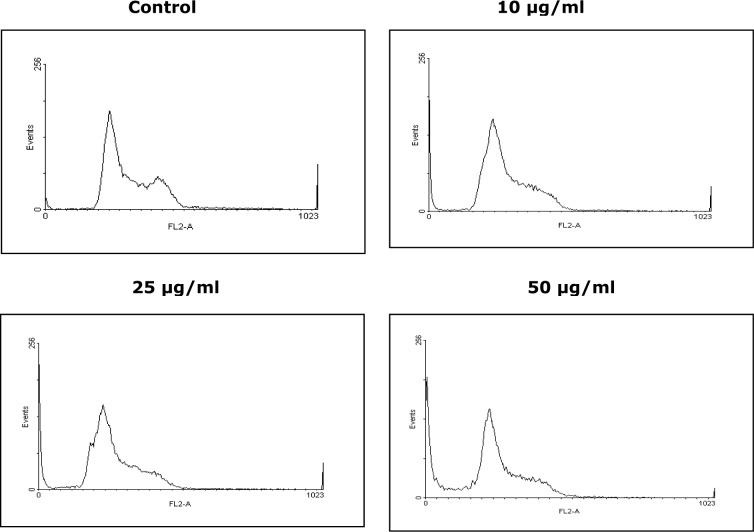
Cells were seeded at 1-2×10^6^ cells in 12 well plates and incubated for 2 h, then treated with (10, 25 and 50 μg/mL) concentrations of PREO for 24 h. Stained cells were protected from light until they were evaluated on flow cytometry, which was performed using an Epics XL-MCL flow cytometer.

In order to investigate the changes in cell cycle regulation, the KBM-5 cells were treated with different concentrations of PREO i.e., 10, 25 and 50 µg/mL for 24 h and subjected to FACS analysis. Treatment with PREO showed a significant increment in the percentage of cells in the G0/G1 phase and proportion of cells in S phase was also increased with time (Figure 4(Fig. 4)). Similar to present investigation, the *P. massoniana* bark extract also induced apoptosis in Hep-G2 cells (Ma et al., 2010[38]) and *P. koraiensis* essential oil induced G1-arrest and inhibits cell proliferation and migration in human colon cancer cells (Cho et al., 2014[14]). Thus, present study revealed that *Pinus* plant influences the cell cycle and induced apoptosis.

### Pinus roxburghii inhibits TNF-α induced NF-κB activation in a dose-dependent manner

**Figure 5:**
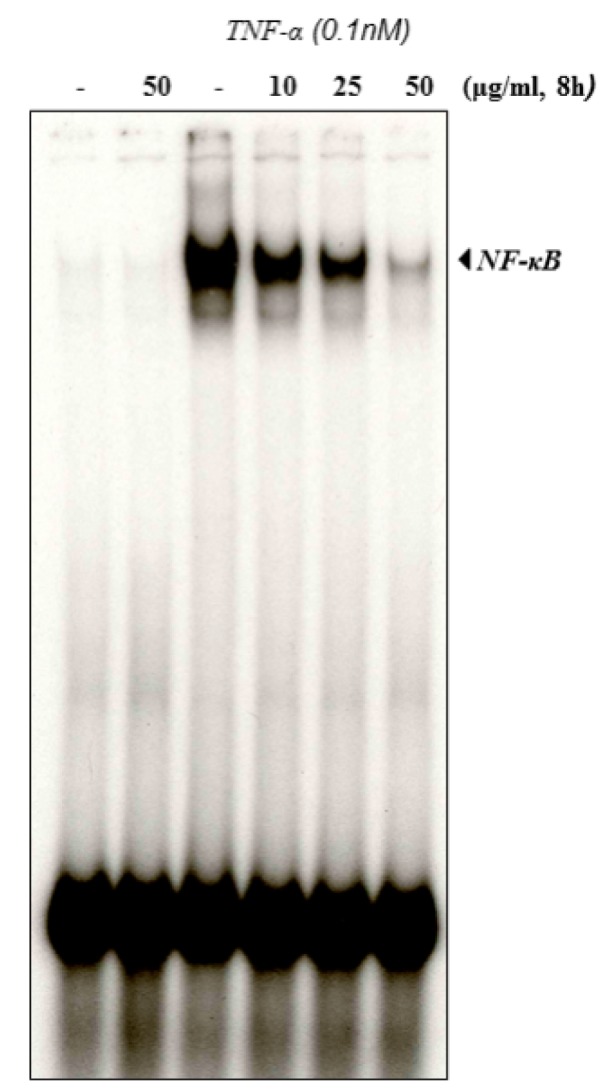
Effect of *Pinus roxburghii *on NF-κB activation. *Pinus roxburghii *suppresses TNF-induced NF-κB activation in a dose-dependent manner. KBM-5 cells (2.0×10^6^ cells/mL) were pre-incubated with PREO (0, 10, 25, 50 μg/mL) for 8 h and then treated with 0.1 nM TNF for 30 min. Nuclear extracts were prepared and assayed for NF-κB activation by EMSA (left panel). Values below the EMSA gel indicate percent growth inhibition. Percent inhibition of NF-κB by PREO was calculated by quantitation of NF-κB bands using a Storm 820 phosphorimager equipped with ImageQuant software (Amersham) (right panel). Data represent the mean±SD of three measurements. *p<0.05, versus control.

The chronic myeloid leukemia cells (KBM-5) were used for TNF-α induced NF-κB activation study since these cells express tumor necrosis factor (TNF) receptors and also inflammatory pathway is well understood (Han et al., 2014[24]). The cells were treated with different concentrations of PREO and activated with TNF-α for NF-κB. As evident from results, the PREO induced TNF-α (NF-κB) in KBM-5 cells, the TNF-α-induced NF-κB activation was dose-dependent to PREO concentration. It was observed that the inhibition in NF-κB activation was maximum at 50 μg/mL dose (Figure 5(Fig. 5)). In support of these findings, the analgesic and anti-inflammatory activity of *P. roxburghii *has also been reported elsewhere (Kaushik et al., 2012[32])*.*

### Pinus roxburghii represses TNF-α-induced NF-κB-dependent gene products associated with survival, proliferation, invasion, and metastasis

**Figure 6:**
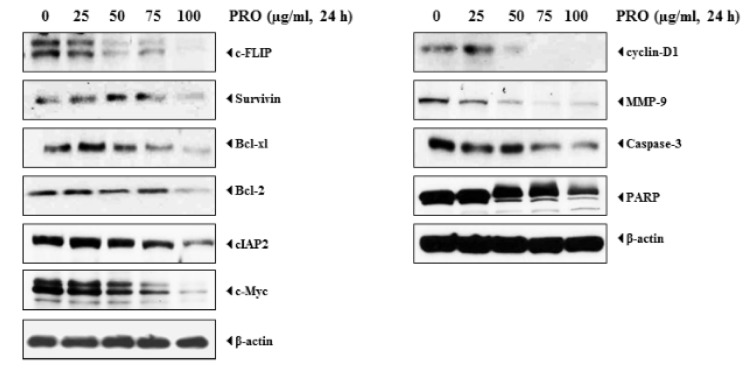
*Pinus roxburghii *suppresses expression of gene products involved in tumor cell survival, proliferation, and metastasis. KBM-5 cells were treated (10, 25, 50 and 100 μg/ml) of PREO for 24 h. Whole cell protein extracts were prepared, separated by electrophoresis, and then transferred to the nitrocellulose membrane. The membrane was sliced according to molecular weight to probe with different antibodies. After stripping, the membrane was reprobed with β-actin to verify equal protein loading.

Since PREO inhibited the survival of cancer cells, also PREO can inhibit the expression of gene products involved in tumor cells survival, which was also investigated. The effect of PREO on anti-apoptotic proteins such as Bcl-2, Bcl-xL, c-IAP-2, and surviving were studied, which are well known to contribute in cell survival. The effect of PREO on cell proliferative proteins i.e., cyclin D1 and c-Myc was investigated, both of which are known to play a major role in cell proliferation. Results revealed that all proteins were down-regulated by increasing the PREO concentration. The effect of PREO on the expression of gene products was higher at a concentration of 100 μg/mL. In next step, effect of PREO was investigated on the activation of caspase-3 and poly (ADP-ribose) polymerase (PARP) cleavage. It was observed that caspase-3 cleaved in a dose-dependent manner. Moreover, PREO also induced PARP cleavage in dose-dependent manner (Figure 6(Fig. 6)). There is lack of reports about the expressions of proteins by PREO. Although, the effect of *P. densiflora* leaf essential oil on expression of caspases, PARP, Bcl-2, Bax and XIAP in YD-8 human oral cancer cells have been reported and a similar trend was observed in the present investigation (Jo et al., 2012[29]).

### GC-MS analysis of P. roxburghii

The PREO was subjected to GC-MS analysis for identification of individual components in PREO. Total fifty-four components were identified constituting 99.47 % of PREO (Table 1(Tab. 1)). The major constituents include α-pinene (27.11 %), which is an important component in *pinus* species (Asta et al., 2006[9]). Other components identified were monoterpenes hydrocarbons 3-carene (9.20 %), β-pinene (7.02 %) and D-limonene (2.33 %). Caryophyllene (2.46 %) and longifolene (0.87 %) sesquiterpenes and oxygenated sesquiterpenes caryophyllene oxide (10.83 %) and humulene epoxide II (2.04 %) were also identified. These results were in good agreement with reported finding i.e., α-pinene was the major component in *P. roxburghii* needles oil having percentage composition of 29.3 % (Iqbal et al., 2011[28]). Some authors also reported the composition of PREO grown in diverse climates (Adams et al., 2014[3]; Hassan and Amjid, 2009[25]). PREO from Algeria showed β-caryophyllene and β-selinene major component (Mimoune et al., 2013[40]). From Nepal, β-caryophyllene was recorded to be a major component (Satyal et al., 2013[46]) and α-pinene, β-pinene, limonene, camphene, betapinene, β-caryophyllene and α-terpinol form India has been reported as major components (Qadir and Shah, 2014[43]). From Egypt, α-pinene, 3-carene, β-pinene and longifolene were reported (Salem et al., 2014[44]). The slight difference in chemical composition of essential oil might be due to climatic, seasonal, geographical or genetic variations (Farooq et al., 2008[17]). However, these findings are in agreement with previous results demonstrating the anti-inflammatory activity of *P. roxburghii *(Kaushik et al., 2012[32]). Plants are a potential sources of drug development of cancer chemoprevention or treatment (Bayala et al., 2014[10]) since plants are potent source of bioactive compounds (Ashraf et al., 2015[4]; Asif, 2015[6]; Aslam et al., 2016[8]; Gull et al., 2015[18]; Hameed et al., 2015[22]; Mensah and Golomeke, 2015[39]). However, a lot of studies are necessary to carry out on the anti-cancer activity of PREO. However, interest in medicinal plant as a source of drug development has increased. However, few researchers reported the cytotoxicity for essential oils (Cavalieri et al., 2004[13]; Lampronti et al., 2006[36]). However, there is a long road before using PREO them as an anticancer agent since very few studies have been done on the PREO. So far, there is need to study the composition of PREO and *in vivo* trials to test the PERO anticancer activity.

## Conclusions

The anticancer, anti-inflammatory activities and chemical composition of PREO was evaluated. Cytotoxicity was evaluated by MTT assay using colon, leukemia, multiple myeloma, pancreatic, head and neck and lung cancer cells. PREO caused cancer cell death and apoptosis at low concentration. PREO inhibited cell proliferation and induced apoptosis in cancer cells and this effect was correlated with the suppression of NF-κB. The TNF-induced inflammation was also inhibited by PREO at very low concentration. On the other hand, the low-dose of PREO did not show cytotoxicity associated with normal physiological function. Therefore, the use of PREO might be a potential candidate as an anticancer agent, which needs to be investigated* in vivo* because p450 enzymes can metabolize the active compound in PREO. Future studies will be focused to identify and purify the bioactive compounds and *in vivo* anticancer activity evaluation. 

## Notes

Arfaa Sajid and Munawar Iqbal (Department of Chemistry, The University of Lahore, Lahore, Pakistan; bosalvee@yahoo.com) contributed equally as corresponding authors.

## Acknowledgements

The authors are grateful to Higher Education Commission Islamabad, Pakistan, for providing grant to conduct this research and Dr. B.B. Aggarwal for providing facilities to carry out this research work in his Cytokine Research Laboratory, Department of Experimental Therapeutics, MD Anderson Cancer Center, Houston, TX, USA. 

## Figures and Tables

**Table 1:**
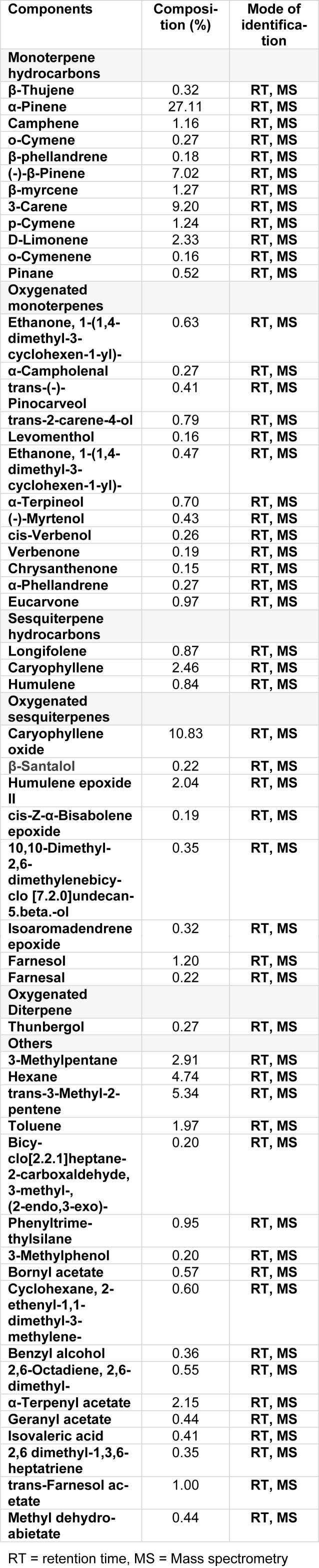
Chemical composition of *Pinus roxburghii* essential oil
